# Cytogenetic Diversity of Simple Sequences Repeats in Morphotypes of *Brassica rapa* ssp. *chinensis*

**DOI:** 10.3389/fpls.2016.01049

**Published:** 2016-07-26

**Authors:** Jin-shuang Zheng, Cheng-zhen Sun, Shu-ning Zhang, Xi-lin Hou, Guusje Bonnema

**Affiliations:** ^1^State Key Laboratory of Crop Genetics and Germplasm Enhancement, Key Laboratory of Biology and Germplasm Enhancement of Horticultural Crops in East China, Ministry of Horticulture, Nanjing Agricultural University, NanjingChina; ^2^Hebei Normal University of Science and Technology, QinhuangdaoChina; ^3^Wageningen UR Plant Breeding, Wageningen University and Research Centre, WageningenNetherlands

**Keywords:** *Brassica rapa*, simple sequence repeats, fluorescence *in situ* hybridization, cytogenetic diversity, heterochromatin

## Abstract

A significant fraction of the nuclear DNA of all eukaryotes is comprised of simple sequence repeats (SSRs). Although these sequences are widely used for studying genetic variation, linkage mapping and evolution, little attention had been paid to the chromosomal distribution and cytogenetic diversity of these sequences. In this paper, we report the distribution characterization of mono-, di-, and tri-nucleotide SSRs in *Brassica rapa* ssp. *chinensis*. Fluorescence *in situ* hybridization was used to characterize the cytogenetic diversity of SSRs among morphotypes of *B. rapa* ssp. *chinensis*. The proportion of different SSR motifs varied among morphotypes of *B. rapa* ssp. *chinensis*, with tri-nucleotide SSRs being more prevalent in the genome of *B. rapa* ssp. *chinensis*. We determined the chromosomal locations of mono-, di-, and tri-nucleotide repeat loci. The results showed that the chromosomal distribution of SSRs in the different morphotypes is non-random and motif-dependent, and allowed us to characterize the relative variability in terms of SSR numbers and similar chromosomal distributions in centromeric/peri-centromeric heterochromatin. The differences between SSR repeats with respect to abundance and distribution indicate that SSRs are a driving force in the genomic evolution of *B. rapa* species. Our results provide a comprehensive view of the SSR sequence distribution and evolution for comparison among morphotypes *B. rapa* ssp. *chinensis*.

## Introduction

Simple sequence repeats (SSRs), also known as microsatellites, are composed of 1–6 nucleotide motifs that are repeated in tandem and are widely and non-randomly distributed in 100–1000s of copies in the genomes of both monocots and dicots (Tautz and Renz, 1984; Toth et al., 2000; Mortimer et al., 2005; Lawson and Zhang, 2006; Hong et al., 2007). Microsatellites are found predominantly in heterochromatin regions, such as centromeric, peri-centromeric, and sub-distal regions of eukaryotic chromosomes (Yang et al., 2005; Li et al., 2010), and sex chromosomes in animals (Beckmann and Weber, 1992; Cuadrado and Jouve, 2011), usually associated with the constitutive heterochromatin (Lohe et al., 1993; Pedersen et al., 1996). Variously non-random, taxon-specific patterns of SSR occurrence call for functional interpretations. Morgante et al. (2002) hypothesized that the relative frequency of microsatellites is higher in the single- or low-copy regions of the genome than in the repetitive regions. Mortimer et al. (2005) showed that SSRs are associated with and around transcribed sequences in *Arabidopsis*. Li et al. (2004) reviewed that SSRs in different positions in a gene can play important roles in regulating its expression and determining the function of its products. The accumulated evidence indicates that SSRs play an important role in chromatin organization, regulation of gene activity (Nagaki et al., 2004), recombination, DNA replication, the cell cycle, the mismatch DNA repair system (Li et al., 2002), and protein coding regions (Sonah et al., 2011).

The fluorescence *in situ* hybridization (FISH) technique has been used to localize one or more SSR loci on chromosomes. FISH has become a strategy for chromosome diagnosis and for investigating plant genome organization (Cuadrado and Schwarzacher, 1998; Cuadrado and Jouve, 2002). SSRs change rapidly during evolution, and thus display polymorphism at homologous sites between closely related species. Begum et al. (2009) showed that the rapid evolution of repetitive DNA sequences has resulted in species-specific repeat variants and the generation of novel repeat families. This characteristic has made SSRs useful as markers in comparative diversity analysis (Zhebentyayeva et al., 2003; Yu et al., 2010; Fang et al., 2013; Zhu et al., 2013) and genetics research (Nanda et al., 1991). SSRs are highly abundant within genomes, they can be widely dispersed or be confined to certain chromosomal regions, and they display a high degree of length polymorphism (Katti et al., 2001; Lawson and Zhang, 2006). Carmona et al. (2013) defined cytogenetic diversity in terms of the differences in abundance and distribution of microsatellites, and also found some specific and motif-dependent hybridization patterns. The repeats AG, AAG, ACT, and ATC presented different *in situ* hybridization patterns that provided cytogenetic landmarks for chromosome identification in barley, *Hordeum vulgare* ssp. *vulgare* (Carmona et al., 2013). Altogether, such variation could be used to determine evolutionary relationships between related species.

*Brassica rapa* belongs to the A genome species group in the Brassicaceae with 2*n* = 20 chromosomes (Nagaharu, 1935), which had a monophyletic origin (Lysak et al., 2005; Cheng et al., 2013). *B. rapa* comprises several sub-species, such as non-heading Chinese cabbage (*B. rapa* ssp. *chinensis*), Chinese cabbage (*B. rapa* ssp. *pekinensis*; Koo et al., 2004), and turnip (*B. rapa* L. ssp. *Rapifera*; Snowdon, 2007). *B. rapa* ssp. *chinensis* is one of the most important leafy vegetable forms in *B. rapa*, and consists of five morphotypes (Pak-choi, Wu ta cai, Cai xin, Fen nie cai, and Tai cai; Viehoever et al., 1920^1^; Gladis and Hammer, 1992; Zheng et al., 2015). The phylogenetic relationships between some *B. rapa* ssp. *chinensis* morphotypes have been determined from morphological, ecological, and molecular data (Yu et al., 2010). In this paper, systematic research was performed to investigate the cytogenetic diversity between intra-specific forms of *B. rapa*.

The objective of the work presented here is to characterize the cytogenetic diversity of SSRs between morphotypes of *B. rapa* ssp. *chinensis* that represent a broad range of cytogenetic diversity. The available genome sequence of Chinese cabbage is an important and fundamental resource for understanding this species^2^ (Wang et al., 2011), but the vast majority of heterochromatic regions remain essentially uncharacterized. The current estimates of SSR frequencies in many organisms differs from reality after comparisons with sequence databases (Hong et al., 2007; Cavagnaro et al., 2010; Gao et al., 2011). The distribution of SSRs in databases has been reported for the Chinese cabbage genome (Hong et al., 2007). In view of their ubiquity and functional importance, detailed information will be necessary to explore the comparative cytogenetics of SSRs in *B. rapa*.

Simple sequence repeats appear to be more abundant in non-coding regions than in coding regions of plant genomes (Hong et al., 2007; Cavagnaro et al., 2010). Tri-nucleotide repeats, the most abundant SSR types in many species (Gao et al., 2003; Shi et al., 2013), were found to be the most frequent in protein coding regions (Sonah et al., 2011). In addition to tri-nucleotide repeats, mono- and di-nucleotide repeats were also predominant in the *B. rapa* genome (Hong et al., 2007; Gao et al., 2011). In this study, we selected mono-, di-, and tri-nucleotide repeats for physical mapping on the chromosomes of *B. rapa*. FISH was performed with mono-, di-, and tri-nucleotides to detect the distributional profile of SSRs and to enhance our understanding of genome organization. The distributional characterization of SSRs revealed a range of cytogenetic diversity that could relate to genome organization, function, and evolutionary trends. We demonstrated that: (1) not all of the SSR-based probes produced FISH signals on all *B. rapa* ssp. *chinensis* chromosomes; (2) some SSR signal intensity did not show a relationship to the abundance in the genome database; (3) the distributional patterns of SSR signals depended on the SSR motif used and the species analyzed; and (4) differences in SSR abundance and density were shown within and between genomes.

## Materials and Methods

### Plant Materials

**FIGURE 1:**
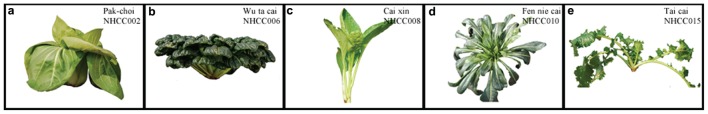
**The five morphotypes of *Brassica rapa* ssp. *chinensis*. (a)** Pak-choi; **(b)** Wu ta cai; **(c)** Cai xin; **(d)** Fen nie cai; **(e)** Tai cai.

Five morphotypes of *B. rapa* ssp. *chinensis*: Pak-choi (cv. NHCC002), Wu ta cai (cv. NHCC006), Cai xin (cv. NHCC008), Fen nie cai (cv. NHCC010), Tai cai (cv. NHCC015) were stored and cultivated at the Key Laboratory of Biology and Germplasm Enhancement of Horticultural Crops in East China, Ministry of Horticulture, China. All of these morphotypes have distinct phenotypic characteristics in terms of leaf shape and size, number of leaves, number of outgrowing axially buds, and flowering time (**Figures 1a–e**).

### Chromosome Preparation

Mitotic metaphase chromosome preparation from root tips followed the procedure described by Zheng et al. (2014). Seeds of all morphotypes were allowed to germinate on moist filter paper in Petri dishes at 25°C until each root was approximately 1.5 cm long. To increase the number of cells at metaphase, seedlings were treated with 2.0 μM 8-oxyquinoline at room temperature for 1.5–2.0 h. After washing three-times for 5 min each in distilled water, the seedlings were fixed in a fresh 3:1 (v/v) mixture of 100% ethanol:glacial acetic acid for 24 h, and preserved in 70% (v/v) ethanol. Root tips were digested with 4% (w/v) cellulase plus 2% (w/v) pectinase for approximately 30 min at 37°C, after which they were squashed in a drop of 45% (v/v) acetic acid. After removing the cover slip by freezing, each slide was air-dried in preparation for FISH.

### Fluorescence *In situ* Hybridization (FISH)

**Table 1** shows all of the synthetic oligonucleotides of 10–20 bp that were used as SSR probes. For mono-nucleotides, the rate of A or C repeats was representative of itself and T and G. The AC/AG motifs represent both themselves and the complementary sequences TG/TC. Two di-nucleotides (AT and GC) were not be used in FISH for their self-complementary structure (Cuadrado et al., 2008). Hybridization with tri-nucleotides (Jurka and Pethiyagoda, 1995), together with mono- and di-nucleotides, were performed on metaphase chromosomes of five representative cultivars of *B. rapa* ssp. *chinensis* morphotypes. The SSR sequences were synthesized by Life Technologies (Nanjing, China), and were labeled with digoxigenin-11-dUTP by random primer labeling followed the manufacturer’s instructions (Roche). The reaction was performed with 2.0 μl of SSR in a 20 μl standard reaction by PCR for 3 h at 37°C, and the reaction was stopped at 65°C for 5 min. Probes were stored at -20°C prior to use in hybridizations.

**Table 1:** The simple sequence repeat (SSR) probes used in this study.

Nucleotide repeat type	SSR probes
Mono-nucleotides	(A)_10_, (C)_10_
Di-nucleotides	(AC)_8_, (AG)_12_
Tri-nucleotides	(GCC)_5_, (ACG)_5_, (ACT)_5_, (CAG)_5_, (CAT)_5_, (CAC)_5_, (ATT)_5_, (AAC)_5_, (AAG)_5_, (AGG)_5_, (AGC)_5_, (ATC)_5_

Fluorescence *in situ* hybridization was performed as described by Zheng et al. (2014). The post-hybridization slide washing procedure was that of Heslop-Harrison (1991). Detection of digoxigenin was performed by incubating the slides in anti-digoxigenin-rhodamine (Roche) at 37°C for 1 h. The chromosomes were then counterstained with 2 μg/μl DAPI (Sigma). Re-probing was performed following the method of Cuadrado and Jouve (1994).

### Image Acquisition and Analysis

Fluorescence *in situ* hybridization signals and images of stained chromosomes were captured using a chilled charge-coupled device (CCD) camera (Axiocam HR, Carl Zeiss, Germany), and images were pseudo-colored and processed using Axiovision software (Carl Zeiss). Detection signals and imaging acquisition were obtained by Zeiss Axio Imager A1 fluorescence microscope. For each SSR motif experiment, we analyzed at least 10 cells with distinct signals. The images from FITC and DAPI staining procedures were recorded separately using a cooled CCD camera. The exposure times depended on the intensity of the signals from each probe. The final images were prepared with Adobe Photoshop, version CS4.

## Results

We used 16 synthetic SSRs as probes for single- and double-target FISH. Differences were observed in the abundance and localization of motifs between the different *B. rapa* ssp. *chinensis* morphotypes, although a general distribution pattern emerged. In all five genomes, most motifs showed a higher density of signal at the centromeric or peri-centromeric regions.

### Distribution of Mono- and Di-nucleotide SSRs in the Genomes of *B. rapa* ssp. *chinensis* Morphotypes

**FIGURE 2:**
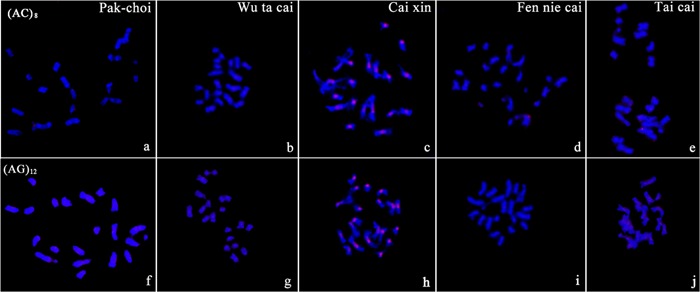
**Characterization of the di-nucleotide repeats AC and AG on chromosomes of *B. rapa* ssp. *chinensis* morphotypes by fluorescent *in situ* hybridization (FISH) using digoxigenin-labeled probes (detected with red rhodamine). (a–e)** (AC)_8_ in Pak-choi, Wu ta cai, Cai xin, Fen nie cai, Tai cai; **(f–j)** (AG)_12_ in Pak-choi, Wu ta cai, Cai xin, Fen nie cai, Tai cai. Chromosomes were counterstained with DAPI.

We did not detect visible signals for the mono-nucleotide repeats A and C on chromosomes of any target genome of *B. rapa* ssp. *chinensis*. The di-nucleotide probes, AC and AG, gave visible signals with different patterns among the five samples (**Figure 2**). Cai xin was the only morphotype in which we detected distinct signals on chromosomes from the two di-nucleotide probes. AG microsatellites showed weak hybridization signals with dispersed patterns in the genomes of Pak-choi and Fen nie cai (**Figures 2f,g**).

### Physical Characterization of Tri-nucleotide Repeats in *B. rapa* ssp. *chinensis* Morphotypes

#### Chromosomal Localization Tri-nucleotide Repeats in Pak-choi

**FIGURE 3:**
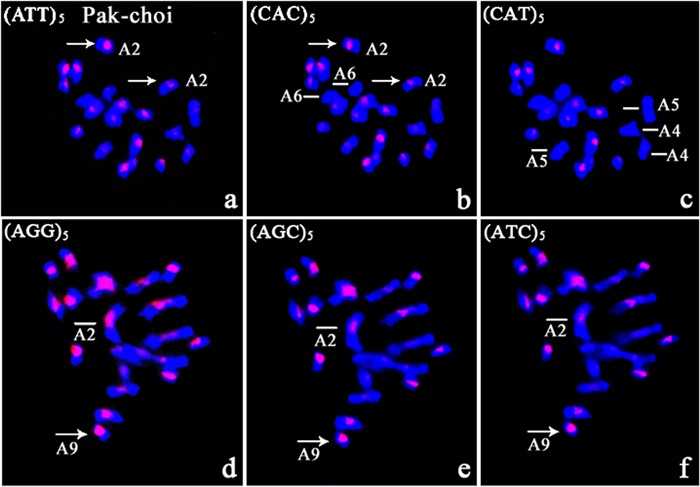
**FISH with CAC and ATT repeat probes on metaphase chromosomes of Pak-choi following *in situ* hybridization with digoxigenin-labeled probes (detected with red rhodamine) and DAPI counterstaining. (a–c)** (ATT)_5_, (CAC)_5_, and (CAT)_5_ in metaphase chromosomes of Pak choi; *arrows* indicate the different fluorescent sites from (ATT)_5_ and (CAC)_5_, and *lines* indicate no signal on chromosome A6 from (CAC)_5_, chromosomes A4 and A5 from (CAT)_5_; **(d–f)** (AGG)_5_, (AGC)_5_, and (ATC)_5_ in metaphase chromosomes of Pak choi; *arrows* indicate the different fluorescent signal sites, and *lines* indicate different intensity fluorescent signals for the SSR loci.

Metaphase chromosomes of five *B. rapa* ssp. *chinensis* morphotypes were hybridized by re-probing preparations with tri-nucleotide repeat probes (**Supplementary Figures S1–S3**). All microsatellite motif probes gave *in situ* hybridization signals on metaphase chromosomes of Pak-choi. Well-defined hybridization signals were produced by ATT, CAC, CAT, AGG, AGC, and ATC probes, and these sequences showed characteristic, motif-dependent distribution patterns (**Figure 3**). The ATT and CAC probes revealed weak hybridization signals restricted to the centromeres of chromosomes A4 and A5, and the CAT repeat probe gave no signal (**Figures 3a–c**). In addition, no signal was detected on chromosome A6 after hybridization with CAC, CAT, and ATT repeat probes. The AGC microsatellites co-localized with ATC repeats on Pak-choi metaphase chromosomes with similar intensity, but showed less intensity than AGG repeats (**Figures 3d–f**). AGG, AGC, and ATC probes showed obvious differences in signal intensity on chromosome A2, AGG being the most intense. These three SSR clusters differed extensively on chromosome A9. AGC and ATC repeats were confined to centromeric regions of this chromosome (**Figures 3e,f**); however, AGG repeats comprised nearly the entire length of the short arm of A9 (**Figure 3d**).

#### Chromosomal Localization Tri-nucleotide Repeats in Wu ta cai

**FIGURE 4:**
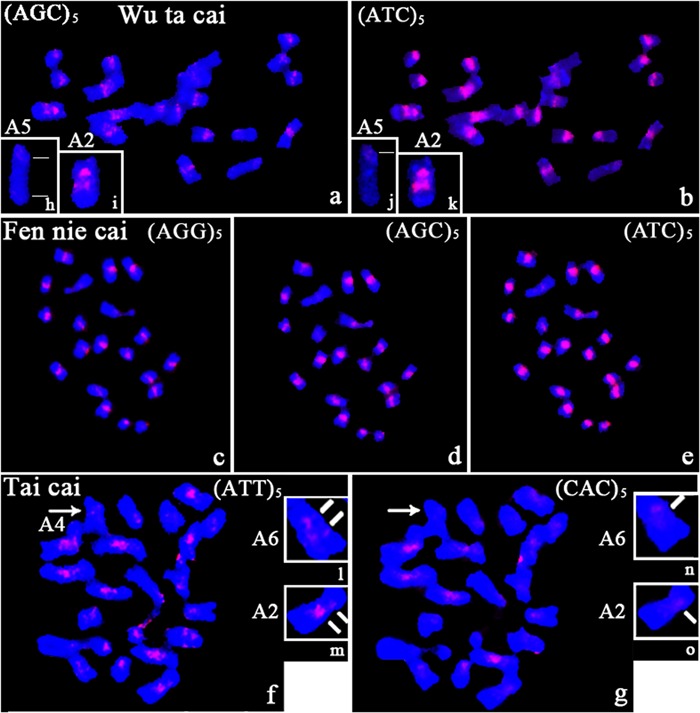
**Photomicrographs showing the distribution of several tri-nucleotide SSRs (AGG, AGC, and ATC) on metaphase chromosomes of Wu ta cai, Fen nie cai, and Tai cai. (a,b)** in Wu ta cai; **(c–e)** in Fen nie cai; **(f,g)** in Tai cai; **(h–k)** variation in the number of signal sites in Wu ta cai; **(l–o)** variation in the number of signal sites in Tai cai; *lines* indicate the number of fluorescent signals in Wu ta cai and Tai cai; *arrows* indicate different intensity fluorescent signals. Chromosomes were counterstained with DAPI.

Differences in the presence/absence and intensity of the hybridization signals were observed on chromosomes of Wu ta cai after hybridization with tri-nucleotide repeat probes (**Figures 4a,b**). All tri-nucleotide repeats were near the centromere on some chromosomes. Polymorphic intercalary signals were observed in two regions of chromosome A2 after hybridizing with AGC and ATC clusters (**Figures 4i,k**). Hybridization signals of differing intensity were obtained, depending on the SSR motif probe. ATC and AGC, which were present on all chromosomes, showed obvious differences in intensity, the former being more intense. A weak signal on the long arm of chromosome A5, after hybridization with and AGC repeat probe, was observed only after increasing the exposure time of the CCD (compare **Figures 4h** and **4j**).

#### Chromosomal Localization Tri-nucleotide Repeats in Cai xin

**FIGURE 5:**
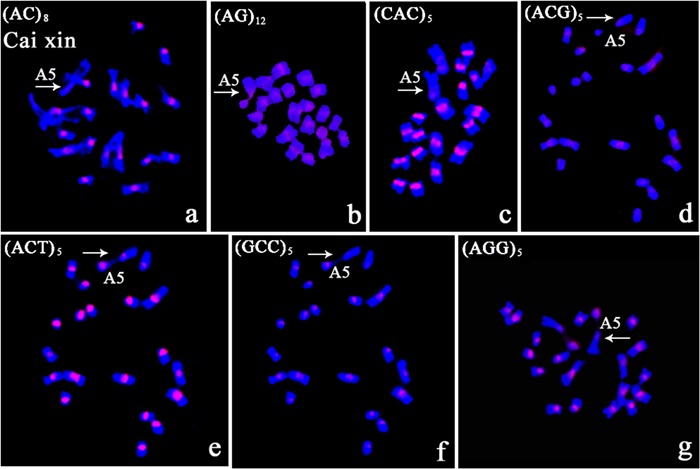
**The polymorphic characterization of the chromosome bearing the NOR in Cai xin following *in situ* hybridization with digoxigenin-labeled probes (detected with red rhodamine) and DAPI counterstaining. (a)** (AC)_8_; **(b)** (AG)_12_; **(c)** (CAC)_5_; **(d)** (ACG)_5_; **(e)** (ACT)_5_; **(f)** (GCC)_5_; **(g)** (AGG)_5_; *arrows* indicate chromosome A5; no signal was detected on the NOR in **(a,d)**; fluorescent signal on the NOR in **(c)**; weak centromeric signal in **(d)**; signals on the NOR and the centromere in **(b,e,g)**.

Variation in intensity and location of the *in situ* signals were observed on metaphase chromosomes of Cai xin. The signals were confined to the centromere for all tri-nucleotide repeats with differing intensities. Polymorphism in terms of presence/absence was observed in the nucleolus organizing region (NOR). NOR signals were generated after hybridization with AG, CAC, ACT, GCC, and AGG repeats (**Figures 5b,c,e–g**). Visible centromeric signals were observed on chromosome A5 for AG, ACG, CAC, and ACT repeat probes (**Figures 5b–e**), but not for AC, GCC, and AGG repeats (**Figures 5a,f,g**).

#### Chromosomal Localization Tri-nucleotide Repeats in Fen nie cai

No specific clusters of SSR-specific signals were observed for tri-nucleotide repeats on chromosomes of Fen nie cai. Polymorphism in terms of presence/absence and intensity of the hybridization signals were observed for some SSRs. The absence signal was revealed from ACT and CAG clusters (**Supplementary Figures S1n,s**). However, signals of varying intensity were observed on the homologous chromosomes after hybridization with AGG, AGC, and ATC repeat probes (**Figures 4c–e**).

#### Chromosomal Localization Tri-nucleotide Repeats in Tai cai

The most intense and rich patterns of *in situ* hybridization signals were produced by ACG, ACT, and ATT repeat probes on chromosomes of Tai cai, and were confined to the peri-centromeric regions. No specific clustered sites were observed for GCC and AAC repeats (**Supplementary Figures S1e** and **S3t**). AAT and CAC repeats were confined around centromeric regions. The ATT repeat probes gave more dispersed and intense centromeric signals, clustered in centromeric and peri-centromeric regions, than did the CAC repeats (**Figures 4f,g**). Variations in the number of signal sites were observed on chromosomes A2 and A6. An intercalary signal was observed on the long arms of chromosome A2 (compare **Figures 4l** with **4n**) and A6 (compare **Figures 4m** with **4o**) from hybridization with ATT repeats, in contrast to only one centromeric signal from CAC repeats (**Figures 4n,o**). No signals were observed from hybridization with a CAC repeat probe on chromosomes A4, A5, and the NOR, but the ATT repeat probe gave weak signals (**Figures 4f,g**).

## Discussion

Rogan et al. (2001) showed that base composition, probe length, and chromosomal location contribute to hybridization signal intensity in FISH. In the present study, all probes ranged from 15 to 20 bp in length. The hybridization patterns obtained depended on the base composition of the probes used and chromosomal location. The synthetic SSR probes were labeled by the random primer method, compared with end labeling, which improved the resolution of SSR loci in FISH (Bouilly et al., 2008). Different SSR probes of the same lengths gave different signal intensities, indirectly reflecting the influence of the target size and the copy numbers of the repeat sequences. The SSR probes gave specific hybridization patterns that provide cytogenetic landmarks for chromosome identification.

### Different SSR Distribution Patterns on Chromosomes Depend on the Species Analyzed

Sonah et al. (2011) showed that mono-, di-, and tri-nucleotide repeats compose the major proportion of SSRs in plant genomes. For tri-nucleotides, A/T-rich repeats (e.g., AAC/GTT, AAG/CTT, and AAT/ATT) were predominant in dicot species. In the monocot barley, however, repeated AAT SSR motifs gave poor hybridization signals (Cuadrado and Jouve, 2007b). Morgante et al. (2002) found that GCC repeats accounted for half of the tri-nucleotide repeats in rice, whereas they were rare in dicots. Lawson and Zhang (2006) examined the most common mono- (A/T) and di-nucleotide (AT and AG) repeats in the *Arabidopsis* genome, and found that polyA/T repeats were predominant, while polyC/G repeats were rare (Sonah et al., 2011). The distribution of AC and AG repeats were linked to the euchromatic and heterochromatic genomic regions, respectively (Cuadrado and Jouve, 2007a). The di-nucleotide AG repeats were located on all chromosomes in *Dendrobium aphyllum* and *D. aggregatum* (Begum et al., 2009), but were exclusively concentrated at the centromeres in *Triticum* (Carmona et al., 2013). The most abundant repeat motifs were A (28.8%), AG (15.4%), AT (13.7%), and AAG (13.3%) clusters, reflecting the A/T rich nature of the *B. rapa* genome (Hong et al., 2007). In this study, nearly all the di- and tri-nucleotide SSRs were detected on metaphase chromosomes of Cai xin and Pak-choi, inferring that these two morphotypes have more types and increased abundance of SSRs compared to the other three morphotypes (Wu ta cai, Fen nie cai, and Tai cai).

Various types of SSR motifs display taxon-specific patterns in the genomes of prokaryotes and eukaryotes (Toth et al., 2000). For example, the most intense hybridization signals were produced by the AGG, AAG, and AAC tri-nucleotide probes in barley (Cuadrado and Jouve, 2007b). The AAC clusters showed the same distribution patterns as AAG repeats in wheat (Cuadrado and Schwarzacher, 1998). AAG repeat units are major contributors to the genomes of dicots (Sonah et al., 2011); they are preferentially associated with peri-centromeric heterochromatin in *Hordeum* species (Carmona et al., 2013), and generally reflect the distribution of heterochromatin and the C-banding pattern in wheat (Cuadrado and Jouve, 1994). AAC repeats are organized in a more dispersed manner, with centromeric regions being largely excluded in chickpea and tomato (Gortner et al., 1998; Gindullis et al., 2001). Some repeats, such as CAG, CAC, and ACG, have specific hybridization sites restricted to the centromeres of metaphase chromosomes in barley (Cuadrado and Jouve, 2007b).

Despite the stable chromosome number and similarities in chromosome size and morphology, differences in numbers and distribution of SSR blocks have been observed among *B. rapa* ssp. *chinensis* morphotypes. The ATT repeat probe gave two signals with different intensities on chromosomes A2 and A6 of Tai cai, indicating that this SSR experienced chromosome-specific accumulation during evolution, as did AGG and ATC repeats in Wu ta cai. In addition, several SSRs (ATT, CAC, AGG, AGC, and ATC) also showed chromosome-specific signals on marker chromosomes of Pak-choi. These three morphotypes may have resulted from chromosomal recombination during speciation and development.

### The Different Distribution Patterns on Chromosomes Depend on the SSR Probes Used

A negative correlation has been observed between repeat numbers and the total length of repeat units in both monocots and dicots (Cavagnaro et al., 2010; Gao et al., 2011; Sonah et al., 2011). Mono-nucleotide repeats of A are the most abundant repeats among all SSRs analyzed (Gao et al., 2011); however, no visible signal was generated, partly implying their dispersed distribution along chromosomes, or may be related to the centromeric function (Cuadrado and Jouve, 2007a). In this work, the di-nucleotide repeats AC and AG only showed distinct *in situ* hybridization signals on chromosomes of Cai xin, although there were weak signals in Pak-choi and Fen nie cai for AG repeats. In addition, three tri-nucleotide repeat probes (AAG, AGG, and AGC) gave distinct signals on all analyzed genomes. Di-nucleotide AG and tri-nucleotide AAG repeats are relatively abundant in the *B. rapa* genome (Hong et al., 2007). AG-rich microsatellites might be the most prevalent SSRs that cluster around centromeric regions in *B. rapa* ssp. *chinensis* morphotypes.

We found that some tri-nucleotide repeats distinctly co-localized within the same genome, although the signals differed in intensity, which could be explained by their intermixed structure at the given loci. Some SSR probes gave weak or dispersed signals at the same physical position, which could possibly be due to closely linked blocks of repeated sequences. Here, we show that individual SSRs vary widely in their relative proportions at the chromosome level, and that the distribution of SSRs along the chromosomes is non-random. The characteristic patterns of SSR distribution show that centromeric regions are more densely populated than the central regions (Rogan et al., 2001). The similar distribution patterns of some SSR motifs indicate that long stretches of different SSRs are of functional importance, and could possibly represent an ancient component of plant genomes (Cuadrado et al., 2000; Cuadrado and Jouve, 2007b). These results are supported by evidence showing that microsatellites display relatively uniform coverage in the genome, and that there are taxon-specific distribution patterns among *B. rapa* ssp. *chinensis* morphotypes. Our results suggest that SSR repeats are the major component of the satellite DNA fraction, and show evolutionary conservation among *B. rapa* ssp. *chinensis* morphotypes.

### Relationship between SSRs and Centromeres

In most higher eukaryotic organisms, chromosomal centromeres are composed of long arrays of satellite repeat sequences and retro-transposons (Henikoff et al., 2001; Jiang et al., 2003). The centromeric and distal regions play important roles during mitosis and meiosis (Li et al., 2002; Hong et al., 2006), and highly divergent sequences are present in the peri-centromeric regions (Wang et al., 2012). The repetitive DNA sequences frequently form clusters within heterochromatin blocks, which are predominantly concentrated at peri-centromeric regions and have been detected in plants with small and compact genomes (Cuadrado and Jouve, 2010; Falistocco and Marconi, 2013). In the present work, most *in situ* SSR signals were confined to centromeric or adjacent regions on *B. rapa* ssp. *chinensis* chromosomes. This distributional characterization may be related to their effect on DNA replication, chromatin organization, and the cell cycle.

### Evolutionary Trends of SSRs among *B. rapa* ssp. *chinensis* Genomes

The relationship between microsatellites and chromosomal evolution has not been clearly documented. The frequency of repeats decreases exponentially with their length, type, and number of SSR motif repeats. This characterization appears to be more conservative in coding than non-coding sequences (Cavagnaro et al., 2010; Sonah et al., 2011; Shi et al., 2013), and less pronounced for di-nucleotides compared to longer repeat types (Cavagnaro et al., 2010). The trends for various repeat types are similar between different chromosomes within the same genome, but the density of repeats may vary between different chromosomes in the same species (Schafer et al., 1986; Katti et al., 2001). Sonah et al. (2011) found species-specific accumulation of particular motif repeats. Variation is present between related species in terms of the abundance and chromosomal distribution of SSR clusters among morphotypes. Schmidt and Heslop-Harrison (1996) demonstrated that microsatellites, representing a substantial fraction of the genome, showed chromosome-specific amplification in plants. High levels of polymorphism and heterozygosity between homologs, in terms of the distribution of AAG and AAC repeats, was shown for out-breeding species in the *Secale strictum* species complex (Cuadrado and Jouve, 2002).

Our results suggest that SSR sequences are more predisposed to being amplified or deleted as a result of independent events. The balance among SSR sequences generated by strand-slippage replication, or recombination and repair mechanisms, cannot be the only explanation for the observed differences in their chromosomal distributions. Another possible explanation would involve selection pressure or mutation (Li et al., 2002). The different chromosomal positions of SSRs involved in the regulation of gene expression (Lawson and Zhang, 2006; Gao et al., 2011), could indicate their underestimated roles in genome evolution (Cuadrado et al., 2008).

The microsatellite sequences analyzed here showed similar chromosome distribution polymorphism patterns, inferring that these SSR loci may result from convergent evolution. However, they differed in intensity or position, indicating that microsatellite repeats can contract or expand over a very short evolutionary time frame (Iwata et al., 2013). The wide distribution of SSRs, and the fact that their positions are restricted to chromosomal centromeres, as revealed by FISH, suggested a general model for the parallel chromosome evolution of repeat-rich heterochromatin in *B. rapa* ssp. *chinensis*.

Carmona et al. (2013) suggested that changes in the amount and distribution of tandem repetitive DNA sequences are major driving forces of genome evolution and speciation. The different regions are thought to undergo different selection pressures (Morgante et al., 2002), which might account for different motif preferences and frequencies among chromosomes. The evolutionary dynamics of microsatellites is generally consistent with plant divergence and evolution (Shi et al., 2013), and the distribution of microsatellites is related to the history of genome evolution and selective constraints (Morgante et al., 2002). The variation in SSRs at the chromosome level may be the result of adaptive divergence, or selection resulting from the stress response among species and populations (Cavagnaro et al., 2010; Gao et al., 2011; Carmona et al., 2013). Whether SSRs are under selection or are neutral as has been reported (Ellegren, 2004), and can be used for exploring the dynamics of the evolutionary process (Santos et al., 2010), will require study.

## Author Contributions

S-nZ and J-sZ: Designed the experiment. X-lH and GB: Provided the materials. J-sZ and C-zS: Performed the experiment. J-sZ: Analyzed the data and wrote the manuscript.

## Conflict of Interest Statement

The authors declare that the research was conducted in the absence of any commercial or financial relationships that could be construed as a potential conflict of interest. The reviewer ST and handling Editor declared their shared affiliation, and the handling Editor states that the process nevertheless met the standards of a fair and objective review.

## Footnotes

1 http://www.plantnames.unimelb.edu.au/Sorting/Brassica_rapa.html#praecox

2 http://brassica.bbsrc.ac.uk/brassica_genome_sequencing_concept.htm

## Supplementary Material

FIGURE S1 **Photomicrographs showing the distribution of the tri-nucleotide repeats (GCC)_5_, (ACG)_5_, (ACT)_5_, (CAG)_5_ on metaphase chromosomes of five *B. rapa* ssp. *chinensis* morphotypes after *in situ* hybridization with digoxigenin-labeled probes (detected with red rhodamine) and DAPI counterstaining. (a,f,k,p)** in Pak-choi; **(b,g,l,q)** in Wu ta cai; **(c,h,m,r)** in Cai xin; **(d,i,n,s)** in Fen nie cai; **(e,j,o,t)** in Tai cai.

FIGURE S2 **Photomicrographs showing the distribution of (AAG)_5_, (AGG)_5_, (AGC)_5_, and (ATC)_5_ repeats on metaphase chromosomes of five *B. rapa* ssp. *chinensis* morphotypes after *in situ* hybridization with digoxigenin-labeled probes (detected with red rhodamine) and DAPI counterstaining. (a,f,k,p)** in Pak-choi; **(b,g,l,q)** in Wu ta cai; **(c,h,m,r)** in Cai xin; **(d,i,n,s)** in Fen nie cai; **(e,j,o,t)** in Tai cai.

FIGURE S3 **Photomicrographs showing the distribution of (CAT)_5_, (CAC)_5_, (ATT)_5_, and (AAC)_5_ repeats on metaphase chromosomes of five *B. rapa* ssp. *chinensis* morphotypes after *in situ* hybridization with digoxigenin-labeled probes (detected with red rhodamine) and DAPI counterstaining. (a,f,k,p)** in Pak-choi; **(b,g,l,q)** in Wu ta cai; **(c,h,m,r)** in Cai xin; **(d,i,n,s)** in Fen nie cai; **(e,j,o,t)** in Tai cai.
