# Supplementary figures and images for: Cytogenetic Diversity of Simple Sequences Repeats in Morphotypes of Brassica rapa ssp. chinensis
Source: Front Plant Sci. 2016 Jul 26;7:1049. doi: 10.3389/fpls.2016.01049 (PMC4961004; doi:10.3389/fpls.2016.01049)

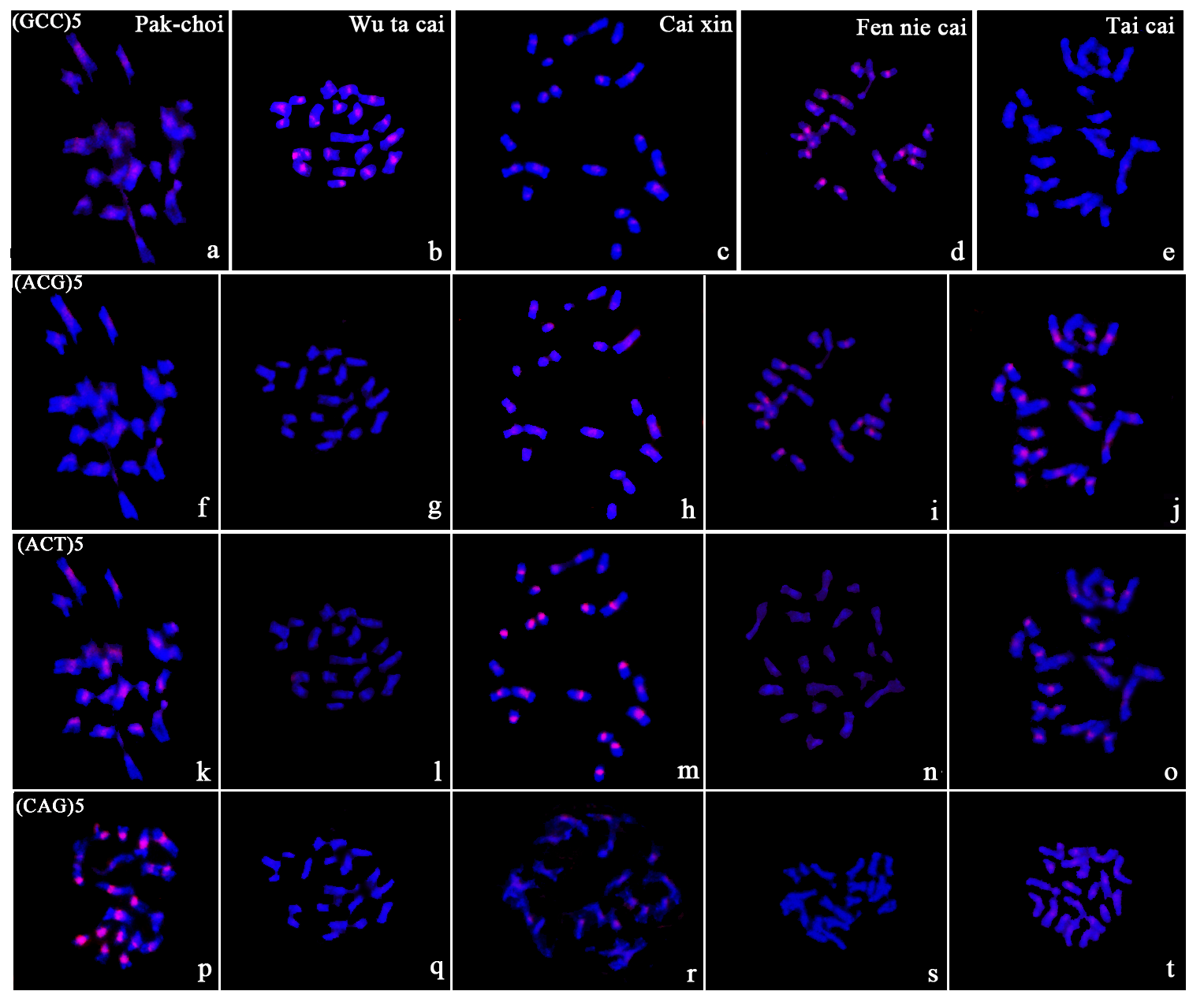

Supplement: FIGURE S1 — Photomicrographs showing the distribution of the tri-nucleotide repeats (GCC)5, (ACG)5, (ACT)5, (CAG)5 on metaphase chromosomes of five B. rapa ssp. chinensis morphotypes after in situ hybridization with digoxigenin-labeled probes (detected with red rhodamine) and DAPI counterstaining. (a,f,k,p) in Pak-choi; (b,g,l,q) in Wu ta cai; (c,h,m,r) in Cai xin; (d,i,n,s) in Fen nie cai; (e,j,o,t) in Tai cai. [file Image_1.TIFF]

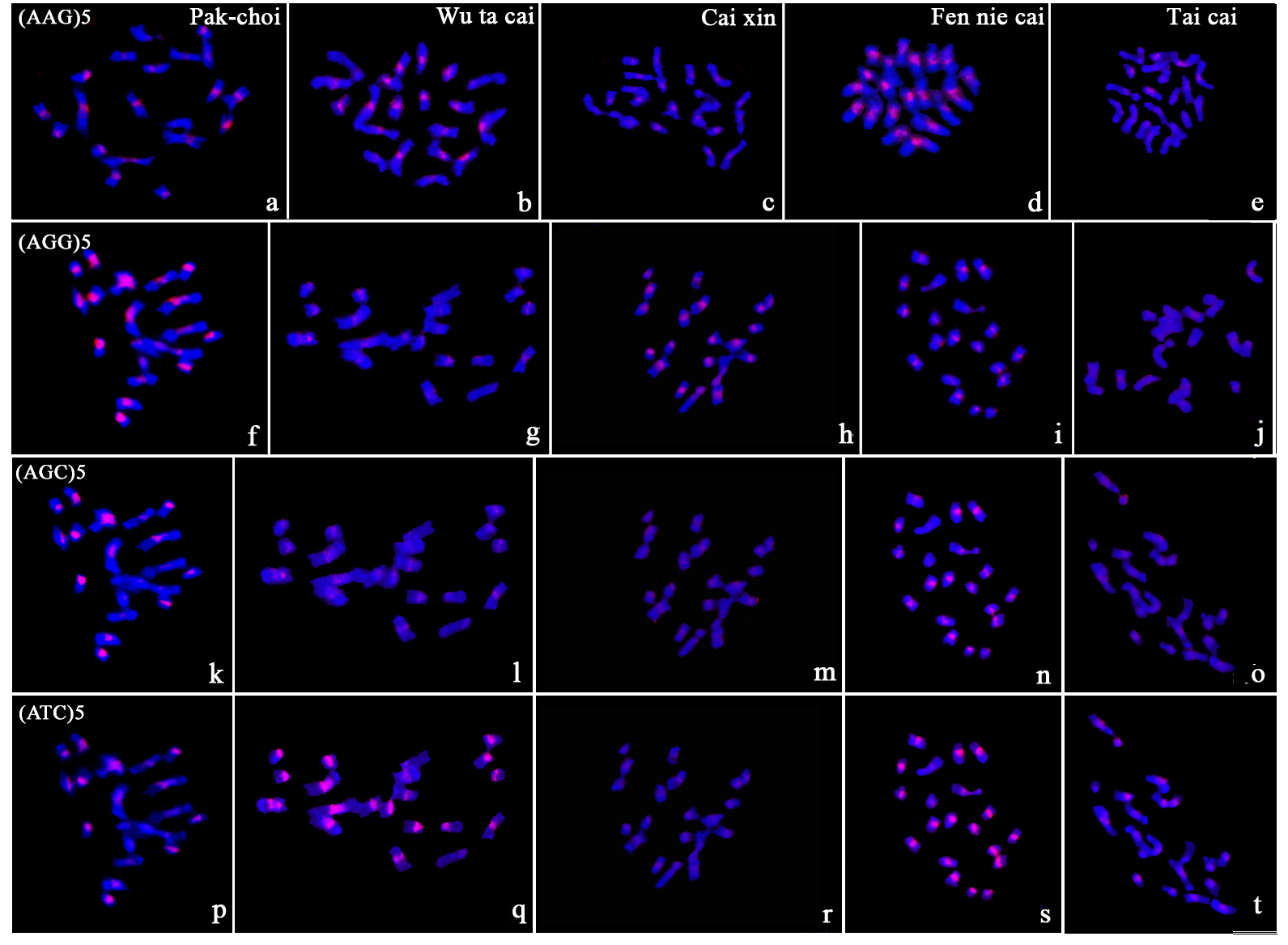

Supplement: FIGURE S2 — Photomicrographs showing the distribution of (AAG)5, (AGG)5, (AGC)5, and (ATC)5 repeats on metaphase chromosomes of five B. rapa ssp. chinensis morphotypes after in situ hybridization with digoxigenin-labeled probes (detected with red rhodamine) and DAPI counterstaining. (a,f,k,p) in Pak-choi; (b,g,l,q) in Wu ta cai; (c,h,m,r) in Cai xin; (d,i,n,s) in Fen nie cai; (e,j,o,t) in Tai cai. [file Image_2.TIFF]

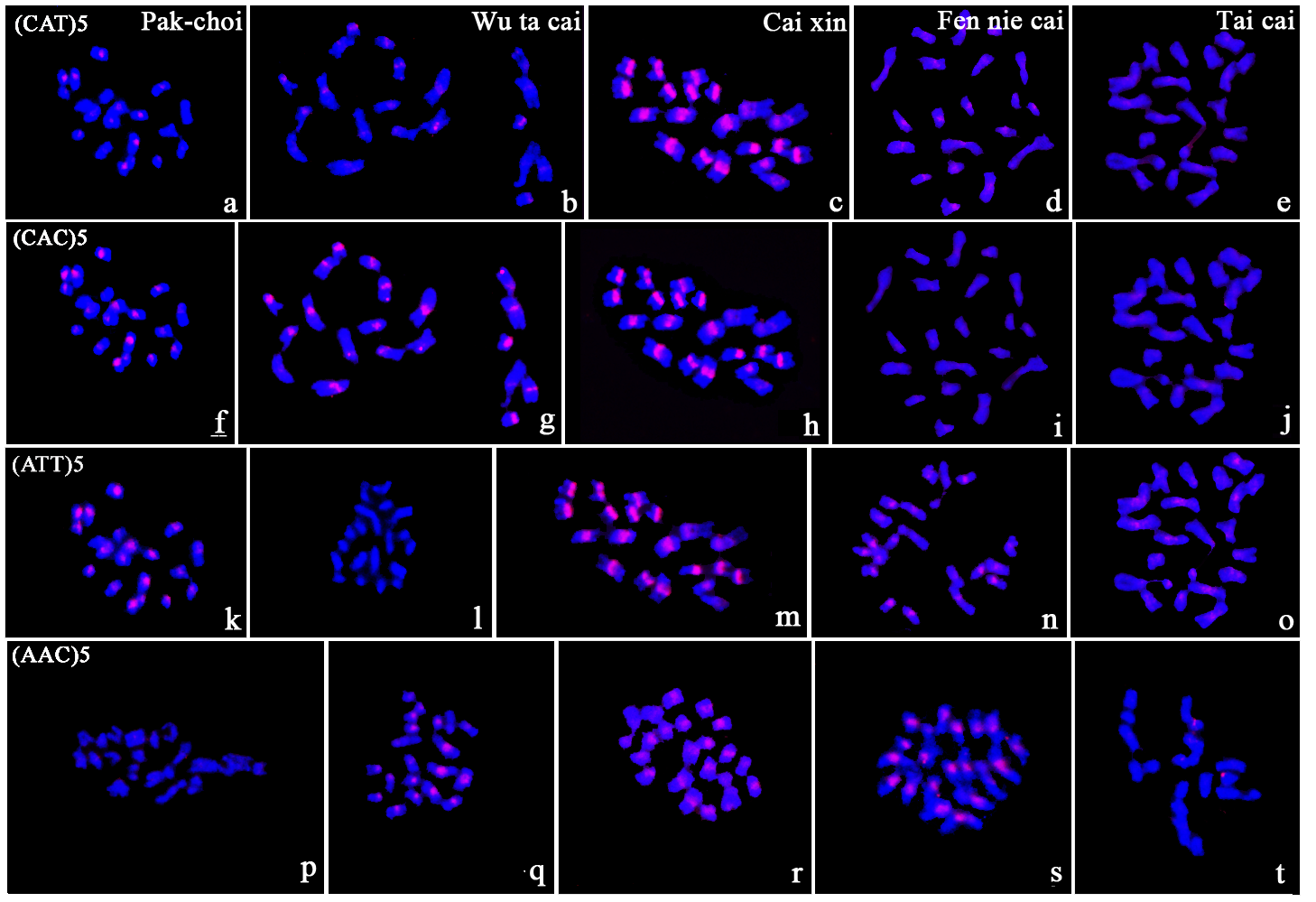

Supplement: FIGURE S3 — Photomicrographs showing the distribution of (CAT)5, (CAC)5, (ATT)5, and (AAC)5 repeats on metaphase chromosomes of five B. rapa ssp. chinensis morphotypes after in situ hybridization with digoxigenin-labeled probes (detected with red rhodamine) and DAPI counterstaining. (a,f,k,p) in Pak-choi; (b,g,l,q) in Wu ta cai; (c,h,m,r) in Cai xin; (d,i,n,s) in Fen nie cai; (e,j,o,t) in Tai cai. [file Image_3.TIFF]
